# Nanoliposomal irinotecan with fluorouracil and folinic acid, FOLFIRINOX, and S-1 as second-line treatment for unresectable pancreatic cancer after gemcitabine/nab-paclitaxel

**DOI:** 10.1038/s41598-024-65689-8

**Published:** 2024-07-23

**Authors:** Taro Shibuki, Taiga Otsuka, Mototsugu Shimokawa, Junichi Nakazawa, Shiho Arima, Masaru Fukahori, Keisuke Miwa, Yoshinobu Okabe, Futa Koga, Yujiro Ueda, Yoshihito Kubotsu, Akitaka Makiyama, Hozumi Shimokawa, Shigeyuki Takeshita, Kazuo Nishikawa, Azusa Komori, Satoshi Otsu, Ayumu Hosokawa, Tatsunori Sakai, Hisanobu Oda, Machiko Kawahira, Shuji Arita, Takuya Honda, Hiroki Taguchi, Kengo Tsuneyoshi, Yasunori Kawaguchi, Toshihiro Fujita, Takahiro Sakae, Kenta Nio, Yasushi Ide, Norio Ureshino, Tsuyoshi Shirakawa, Toshihiko Mizuta, Kenji Mitsugi

**Affiliations:** 1 Division of Drug and Diagnostic Development Promotion, Department for the Promotion of Drug and Diagnostic Development, Translational Research Support Office, National Cancer Center Hospital East, 6-5-1 Kashiwanoha, Kashiwa-shi, Chiba, 277-8577 Japan; 2 Department of Hepatobiliary and Pancreatic Oncology, National Cancer Center Hospital East, 6-5-1 Kashiwanoha, Kashiwa-shi, Chiba, 277-8577 Japan; 3 Department of Medical Oncology, Saga-Ken Medical Center Koseikan, 400 Kase-machi, Saga-shi, Saga, 840-8571 Japan; 4 Department of Internal Medicine, Minato Medical Clinic, 3-11-3 Nagahama, Chuo-ku, Fukuoka-shi, Fukuoka, 810-0072 Japan; 5 Clinical Research Institute, National Kyushu Cancer Center, 3-1-1 Notame, Minami-ku, Fukuoka-shi, Fukuoka, 811-1395 Japan; 6 Department of Biostatistics, Yamaguchi University Graduate School of Medicine, 1-1-1 Minamikogushi, Ube-shi, Yamaguchi, 755-8505 Japan; 7 Department of Gastroenterology, Kagoshima City Hospital, 37-1 Uearata-cho, Kagoshima-shi, Kagoshima, 890-8760 Japan; 8 Digestive and Lifestyle Diseases, Kagoshima University Graduate School of Medical and Dental Sciences, 8-35-1 Sakuragaoka, Kagoshima-shi, Kagoshima, 890-8520 Japan; 9 Division of Gastroenterology, Department of Medicine, Kurume University School of Medicine, 67 Asahi-machi, Kurume-shi, Fukuoka, 830-0011 Japan; 10 Kyoto Innovation Center for Next Generation Clinical Trials and iPS Cell Therapy, Kyoto University Hospital, 54 Kawaharacho, Shogoin, Sakyo-ku, Kyoto, 606-8507 Japan; 11 Multidisciplinary Treatment Cancer Center, Kurume University Hospital, 67 Asahi-machi, Kurume-shi, Fukuoka, 830-0011 Japan; 12 Department of Hepatobiliary and Pancreatology, Saga Medical Center Koseikan, 400 Kase-machi, Saga-shi, Saga, 840-8571 Japan; 13 Department of Hematology and Oncology, Japanese Red Cross Kumamoto Hospital, 2-1-1 Nagamine-Minami, Higashi-ku, Kumamoto-shi, Kumamoto, 861-8520 Japan; 14 Department of Internal Medicine, Karatsu Red Cross Hospital, 2430 Watada,Karatsu-shi, Saga, 847-8588 Japan; 15 Department of Hematology Oncology, Japan Community Healthcare Organization Kyushu Hospital, 1-8-1 Kishinoura, Yahatanishi-ku, Kitakyushu-shi, Fukuoka, 806-8501 Japan; 16 Cancer Center, Gifu University Hospital, 1-1 Yanagido, Gifu-shi, Gifu, 501-1194 Japan; 17 Department of Gastroenterology, Japanese Red Cross Nagasaki Genbaku Hospital, 3-15, Morimachi, Nagasaki-shi, Nagasaki, 852-8511 Japan; 18 Department of Medical Oncology and Hematology, Oita University Faculty of Medicine, 1-1 Idaigaoka, Hasama-machi, Yufu-shi, Oita, 879-5593 Japan; 19 Department of Gastrointestinal Medical Oncology, National Hospital Organization Shikoku Cancer Center, 160 Minamiumemoto-cho, Matsuyama-shi, Ehime, 791-0280 Japan; 20 Department of Clinical Oncology, University of Miyazaki Hospital, 5200, Kiyotakechoukihara, Miyazaki-shi, Miyazaki, 889-1692 Japan; 21 Department of Medical Oncology, National Hospital Organization Kumamoto Medical Center, 1-5, Ninomaru, Chuo-ku, Kumamoto-shi, Kumamoto, 860-0008 Japan; 22 Division of Integrative Medical Oncology, Saiseikai Kumamoto Hospital, 5-3-1,Oumi, Minami-ku, Kmamoto-shi, Kumamoto, 861-4193 Japan; 23 Department of Gastroenterology, Kagoshima Kouseiren Hospital, 1-13-1, Yojirou,Kagoshima-shi, Kagoshima, 890-0062 Japan; 24 Department of Chemotherapy, Miyazaki Prefectural Miyazaki Hospital, Miyazaki, Japan; 25 Department of Gastroenterology and Hepatology, Nagasaki University Graduate School of Biomedical Sciences, 1-7-1 Sakamoto, Nagasaki-shi, Nagasaki, 852-8501 Japan; 26 Department of Gastroenterology, Saiseikai Sendai Hospital, 2-46 Harada-cho, Satsumasendai-shi, Kagoshima, 895-0074 Japan; 27 Department of Gastroenterology, Izumi General Medical Center, 520, Myoujin-cho, Izumi-shi, Kagoshima, 899-0131 Japan; 28 Department of Gastroenterology, Asakura Medical Association Hospital, 422-1, Raiha, Asakura-shi, Fukuoka, 838-0069 Japan; 29 Department of Medical Oncology, Sasebo Kyosai Hospital, 10-17 Shimanji-cho, Sasebo-shi, Nagasaki, 857-8575 Japan; 30 Department of Medical Oncology, Hamanomachi Hospital, 3-3-1 Nagahama, Chuo-ku, Fukuoka-shi, Fukuoka, 810-8539 Japan; 31 Department of Internal Medicine, National Hospital Organization Saga Hospital, 1-20-1 Hinode, Saga-shi, Saga, 849-8577 Japan; 32 Department of Medical Oncology, Kimitsu Chuo Hospital, 1010 Sakurai, Kisarazu-shi, Chiba, 292-8535 Japan; 33 Eikoh Hospital, 3-8-15 Befu-nishi, Shime-machi, Kasuya-gun, Fukuoka, 811-2232 Japan; 34 Clinical Hematology Oncology Treatment Study Group, 1-14-6 Muromi-gaoka, Nishi-ku, Fukuoka-shi, Fukuoka, 819-0030 Japan; 35 Department of Internal Medicine, Fujikawa Hospital, 1-2-6 Matsubara, Saga-shi, Saga, 840-0831 Japan

**Keywords:** Pancreatic cancer, Second line, Nanoliposomal irinotecan, S-1, FOLFIRINOX, Cancer, Diseases, Gastroenterology, Oncology

## Abstract

This study aimed to compare second-line treatment outcomes for patients with unresectable pancreatic cancer previously treated with gemcitabine plus nab–paclitaxel (GnP) therapy. We conducted an integrated analysis of two retrospective studies included 318 patients receiving nanoliposomal irinotecan + 5-fluorouracil/folinic acid (NFF) (n = 102), S-1 (n = 57), or FOLFIRINOX (n = 14) as second-line treatment. Median overall survival (OS) in the NFF group was 9.08 months, significantly better than S-1 (4.90 months, *P* = 0.002). FOLFIRINOX had a median OS of 4.77 months, not statistically different from NFF. Subgroup analyses of OS indicated NFF was generally superior, however, a statistical interaction was observed between the treatment regimen in serum Alb < 3.5 g/dL (*P* = 0.042) and serum CRP ≥ 0.3 mg/dL (*P* = 0.006). Median progression-free survival (PFS) was 2.93 months for NFF, significantly better than S-1 (2.53 months, *P* = 0.024), while FOLFIRINOX had a comparable PFS (3.04 months, *P* = 0.948). Multivariate analysis identified the serum CRP, serum CA19-9, duration of first-line GnP therapy, and use (yes/no) of S-1 for second-line treatment as independent predictors for OS. This study concludes that second-line NFF therapy demonstrated a more favorable OS compared to S-1 therapy, however, it is still important to consider the patient background characteristics while selecting the most appropriate treatment.

## Introduction

Pancreatic cancer is the seventh leading cause of cancer-related death worldwide, and the fourth leading cause of cancer death in Japan^1,2^. Although surgical resection is currently the only available curative treatment for pancreatic cancer, only 15% of pancreatic cancer patients are suitable candidates for curative pancreatectomy, because most patients have either distant metastases or locoregional spread, including vascular invasion, even at diagnosis^3^. Thus, systemic chemotherapy plays a significant role in the treatment of patients with unresectable pancreatic cancer. Significant progress has been made in systemic chemotherapy for patients with metastatic pancreatic cancer with the development of the gemcitabine plus nab-paclitaxel (GnP) and FOLFIRINOX (fluorouracil, leucovorin, irinotecan, and oxaliplatin) regimens^4,5^. However, almost all patients eventually show disease progression, with patients with this cancer showing a dismal 5 year survival of less than 5%^6^. Therefore, effective second-line treatment(s) is necessary to improve the prognosis of patients with unresectable pancreatic cancer.

In a randomized controlled phase III trial (NAPOLI-1), patients with metastatic disease who had previously received gemcitabine-based therapy were found to show longer median overall survival (OS) following second-line treatment with nanoliposomal irinotecan + 5-fluorouracil/folinic acid (NFF) as compared to that with 5-fluorouracil/folinic acid (6.2 vs. 4.2 months; hazard ratio [HR], 0.75; *P* = 0.042). Based on this finding, this regimen is currently recommended by the National Comprehensive Cancer Network (NCCN) guidelines for patients with a good performance status presenting with disease progression after first-line gemcitabine-based chemotherapy^7,8^.

In Japan, GnP is widely used as a first-line chemotherapy regimen for patients with unresectable pancreatic cancer. For patients showing disease progression after GnP therapy, NFF, sequential treatment with S-1, and FOLFIRINOX are clinically available in the second-line setting. In phase II trials, S-1 treatment yielded clinically acceptable outcomes in patients showing disease progression after gemcitabine-based treatment, with reported response rates in the range of 9.5–15% and median OS durations in the range of 4.1–4.5 months^9,10^. Administration of FOLFIRINOX as second-line treatment also yielded clinically meaningful outcomes in patients showing disease progression after first-line gemcitabine-based treatment. One retrospective study conducted in Korea compared NFF and FOLFIRINOX treatment in the second-line setting and reported comparable efficacies^11,12^. As such, S-1 and FOLFIRINOX are also used as second-line treatments besides NFF in daily clinical practice. However, there are no reports of studies comparing these regimens. We conducted this retrospective study to compare the outcomes of treatment with these regimens in the second-line setting in unresectable pancreatic cancer patients presenting with disease progression after first-line GnP therapy.

## Results

### Patients

**Figure 1:**
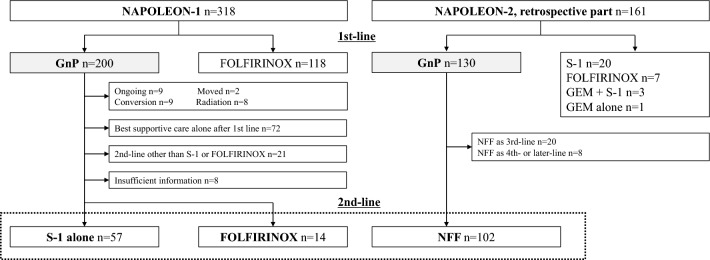
Flow diagram of the study patients. *GnP* gemcitabine plus nab–paclitaxel, *GEM* gemcitabine, *NFF* nanoliposomal irinotecan with fluorouracil folinic acid.

Patients with unresectable pancreatic cancer enrolled in the NAPOLEON-1 and -2 (retrospective part) studies who had received GnP as first-line chemotherapy, followed by NFF, S-1, or FOLFIRINOX as second-line therapy were included in our present analysis (Fig. 1)^13,14^. A total of 173 patients were enrolled between December 2013 and May 2021. By the end of the follow-up period, 99 patients (57.2%) died. The median follow-up period, from the date of initiation of the second-line treatment to the date of the last follow-up, was 4.6 months. The baseline characteristics of the patients are shown in Table 1. There were significant imbalances in the following three variables among the three treatment groups. The S-1 group had a higher rate of a worse ECOG PS than the NFF or FOLFIRINOX group (*P* = 0.040). Patients with liver metastases were more numerous in the NFF group than in the S-1 or FOLFIRINOX group (*P* = 0.007). The percentage of patients with serum CA19-9 < 1000 U/mL was lower in the FOLFIRINOX group than in the NFF or S-1 group (*P* = 0.035). Patients characteristics without multiple imputation are also shown in Supplementary Table 1.

**Table 1:** Baseline characteristics.

	NFF (n = 102)	S-1 (n = 57)	FOLFIRINOX (n = 14)	*P*-value
Age (years), *n* (%)				
< 70	52 (51.0)	32 (56.1)	10 (71.4)	0.335
≥ 70	50 (49.0)	25 (43.9)	4 (28.6)	
Sex (M/F), *n* (%)				
Male	61 (59.8)	34 (59.6)	9 (64.3)	0.946
Female	41 (40.2)	23 (40.4)	5 (35.7)	
ECOG PS, *n* (%)				
0	49 (48.0)	16 (28.1)	7 (50.0)	0.040
1 or more	53 (52.0)	41 (71.9)	7 (50.0)	
Histology, *n* (%)				
Adenocarcinoma	93 (91.2)	50 (87.7)	13 (92.9)	0.513
Others^a^	5 (4.9)	0	0	
Unknown	4 (3.9)	7 (12.3)	1 (7.1)	
Prior pancreatectomy, *n* (%)	14 (13.7)	5 (8.8)	3 (12.4)	0.397
Disease extension, *n* (%)				
Locally advanced	12 (11.8)	14 (24.6)	2 (14.3)	0.108
Metastatic	90 (88.2)	43 (75.4)	12 (85.7)	
Metastatic site, *n* (%)				
Liver	65 (63.7)	22 (38.6)	6 (42.9)	0.007
Lung	16 (15.7)	10 (17.5)	2 (14.3)	0.935
Peritoneal	28 (27.5)	22 (38.6)	5 (35.7)	0.332
Ascites, *n* (%)	18 (17.6)	10 (17.5)	2 (14.3)	0.951
Albumin (g/dL), *n* (%)				
≥ 3.5	60 (58.8)	33 (57.9)	8 (57.1)	0.989
< 3.5	42 (41.2)	24 (42.1)	6 (42.9)	
CRP (mg/dL), *n* (%)				
< 0.3	43 (42.2)	19 (33.3)	6 (42.9)	0.529
≥ 0.3	59 (57.8)	38 (66.7)	8 (57.1)	
CA19-9 (U/mL), *n* (%)				
< 1000	46 (45.1)	33 (57.9)	11 (78.6)	0.035
≥ 1000	56 (54.9)	24 (42.1)	3 (21.4)	
Duration of first-line GnP, n (%)				
≥ 6	64 (62.7)	39 (68.4)	5 (35.7)	0.077
< 6	38 (37.3)	18 (31.6)	9 (64.3)	

*NFF* nanoliposomal irinotecan with fluorouracil and folinic acid, *ECOG PS* Eastern Cooperative Oncology Group performance status, *serum CRP* C-reactive protein, *serum CA19-9* carbohydrate antigen 19-9, *GnP* Gemcitabine plus nab-paclitaxel.

^a^Acinar cell carcinoma, adenosquamous carcinoma and intrapapillary mucinous carcinoma.

### Efficacy results

**Figure 2:**
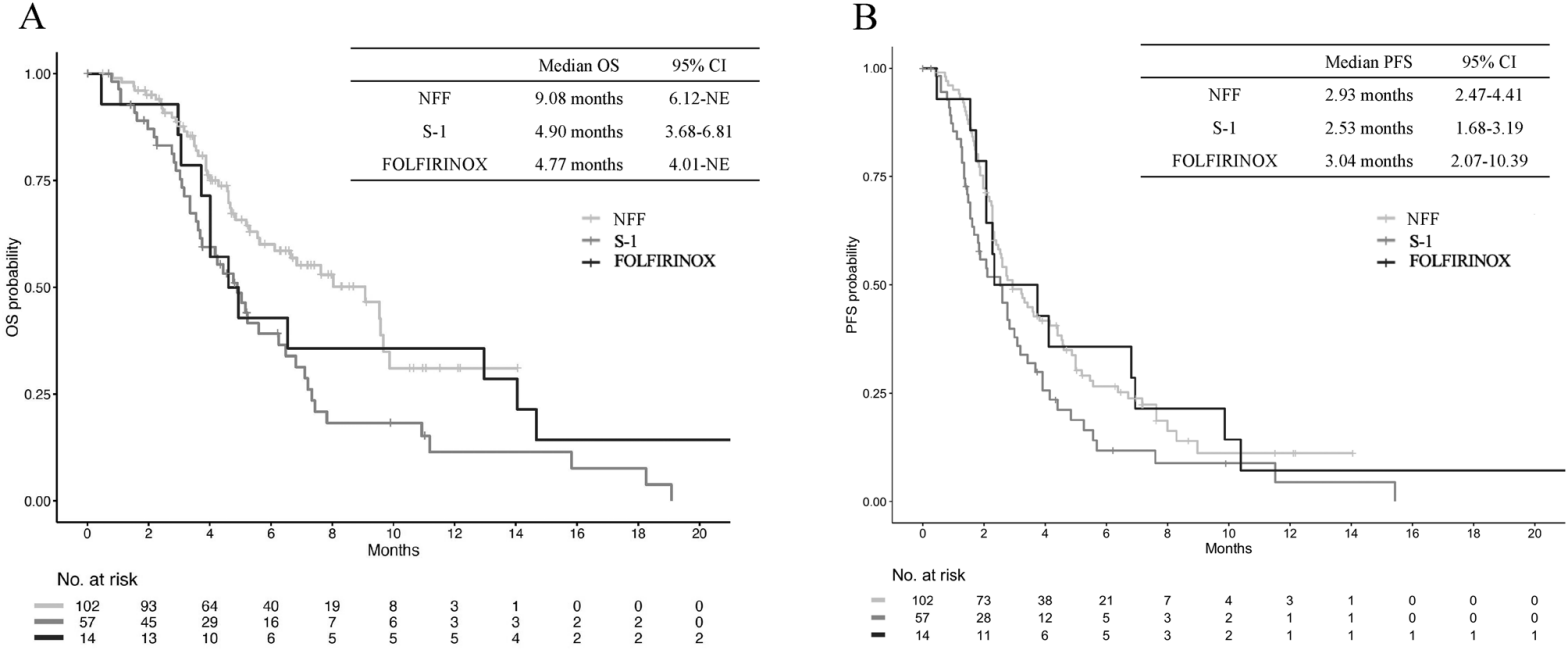
Kaplan–Meier curves of OS (**A**) and PFS (**B**) in patients with unresectable pancreatic cancer receiving second-line chemotherapy. *NFF* nanoliposomal irinotecan with fluorouracil and folinic acid, *OS* overall survival, *CI* confidence interval, *PFS* progression-free survival.

The efficacy results are summarized in Table 2. The objective response (complete response [CR] + partial response [PR]) rate (ORR) was 4.9, 1.8, and 0% in the NFF, S-1, and FOLFIRINOX groups, respectively, with no statistically significant difference in the rate among the three groups. The disease control rate (DCR) in the NFF group was significantly higher as compared with that in the S-1 group (48.0% vs. 21.1%, *P* < 0.001), and comparable to that in the FOLFIRINOX group (vs. 42.9%, *P* = 0.484). The median OS in the NFF group was 9.08 months (95% CI 6.12–Not evaluable [NE]), while that in the S-1 group was significantly worse, at 4.90 months (95% CI 3.68–6.81, unstratified HR 1.97 [95% CI 1.28–3.01], *P* = 0.002). The FOLFIRINOX group showed a median OS of 4.77 months, and the difference between FOLFIRINOX and NFF was not statistically significant (95% CI 4.01–NE, unstratified HR 1.27 [95% CI 0.64–2.48], *P* = 0.484) (Fig. 2A). Similar results were observed for the PFS; the median PFS in the NFF group was 2.93 months (95% CI 2.47–4.41), and that in the S-1 group was significantly worse, at 2.53 months (95% CI 1.68–3.19, unstratified HR: 1.52 [95% CI 1.06–2.17], *P* = 0.024); the median PFS in the FOLFIRINOX group was not significantly different, at 3.04 months (95% CI 2.07–10.39, unstratified HR: 0.98 [95% CI 0.54–1.77], *P* = 0.948) (Fig. 2B).

**Table 2:** Efficacy results.

	NFF (n = 102)	S-1 (n = 57)	FOLFIRINOX (n = 14)	*P*-value
Best overall response, n (%)				
CR	0	0	0	
PR	5 (4.9)	1 (1.8)	0	
SD	44 (43.1)	11 (19.3)	6 (42.9)	
PD	44 (43.1)	33 (57.9)	7 (50.0)	
NE	9 (8.8)	12 (21.1)	1 (7.1)	
ORR (CR + PR), n (%)	5 (4.9)	1 (1.8)	0	0.654
DCR (CR + PR + SD), n (%)	49 (48.0)	12 (21.1)	6 (42.9)	0.002

*NFF* nanoliposomal irinotecan with fluorouracil and folinic acid, *CR* complete response, *PR* partial response, *SD* stable disease, *PD* progressive disease, *ORR* overall response rate, *DCR* disease control rate.

The results of the univariate and multivariate analyses conducted to identify the determinants of the OS are listed in Table 3. The univariate analyses identified a higher ECOG PS, serum Alb < 3.5 g/dL, elevated serum CRP, serum CA19-9 ≥ 1000 U/mL, shorter duration of first-line GnP therapy, and second-line treatment with S-1 as being significantly associated with a shorter OS. Multivariate analysis identified serum CRP, serum CA19-9, duration of first-line GnP therapy, and use (yes/no) of second-line treatment with S-1 as independent determinants of the OS. Similarly, univariate and multivariate analyses were conducted to identify determinants of OS in cohort without multiple imputation, with similar results (Supplementary Table 2).

**Table 3:** Univariate and multivariate analyses conducted using Cox proportional hazards models to predict survival in patients with unresectable pancreatic cancer.

Variables	Univariate analysis			Multivariate analysis		
	HR	95% CI	*P*-value	HR	95% CI	*P*-value
Age						
< 70 years	Reference			Reference		
≥ 70 years	0.937	0.628–1.399	0.751	0.908	0.595–1.388	0.657
Sex						
Female	Reference			Reference		
Male	0.967	0.645–1.448	0.871	0.809	0.522–1.254	0.344
ECOG PS						
0	Reference			Reference		
1 or more	1.876	1.225–2.874	0.004	1.551	0.962–2.501	0.072
Prior pancreatectomy: Yes	0.819	0.425–1.579	0.552	0.758	0.366–1.571	0.457
Metastases: Yes	0.934	0.552–1.581	0.800	1.322	0.754–2.317	0.329
Albumin						
≥ 3.5 g/dL	Reference			Reference		
< 3.5 g/dL	1.831	1.228–2.729	0.003	1.416	0.907–2.212	0.126
CRP						
< 0.3 mg/dL	Reference			Reference		
≥ 0.3 mg/dL	3.291	2.032–5.330	< 0.001	2.670	1.580–4.608	< 0.001
CA19-9						
< 1000 U/mL	Reference			Reference		
≥ 1000 U/mL	1.770	1.175–2.667	0.006	1.948	1.236–3.069	0.004
Duration of first-line GnP						
≥ 6 months	Reference			Reference		
< 6 months	1.974	1.326–2.938	0.002	2.270	1.476–3.492	< 0.001
Second-line treatment						
NFF	Reference			Reference		
S-1	1.966	1.283–3.011	0.002	2.323	1.435–3.492	< 0.001
FOLFIRINOX	1.271	0.650–2.484	0.484	1.267	0.604–2.663	0.537

*HR* hazard ratio, *CI* confidence interval, *ECOG PS* Eastern Cooperative Oncology Group performance status, *LDH* lactate dehydrogenase, *CRP serum* C-reactive protein, *serum CA19-9* carbohydrate antigen 19-9, *GnP* Gemcitabine plus nab-paclitaxel, *NFF* nanoliposomal irinotecan with fluorouracil and folinic acid.

**Figure 3:**
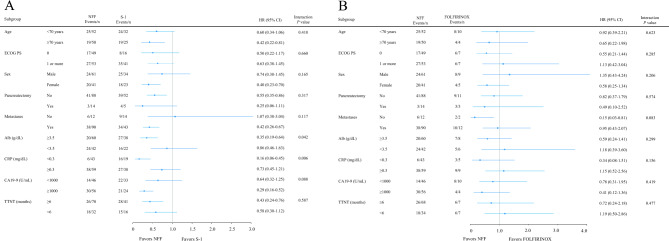
Forest plot of overall survival in NFF vs. S-1 subgroups (**A**), and NFF vs. FOLFIRINOX subgroups (**B**). *NFF* nanoliposomal irinotecan with fluorouracil and folinic acid, *HR* hazard ratio, *CI* confidence interval, *ECOG PS* Eastern Cooperative Oncology Group performance status, *CRP* C–reactive protein, *CA19-9* carbohydrate antigen 19-9, *TTNT* time to next treatment.

Subgroup analyses performed to further explore the effects of the three treatment regimens on the OS are shown in Fig. 3. In most subgroups, NFF treatment was associated with a better OS than S-1 treatment. However, a statistical interaction was observed between the treatment regimen and serum Alb < 3.5 g/dL (P = 0.042) and/or serum CRP ≥ 0.3 mg/dL (*P* = 0.006). There were no significant differences in subgroups between the NFF and FOLFIRINOX groups.

### Safety profile

Safety profile of patients treated with NFF according to UGT1A1 status was shown in Supplementary Table 3.

### Post second-line treatment (Table 4)

**Table 4:** After second-line treatment.

	NFF (n = 79)	S-1 (n = 50)	FOLFIRINOX (n = 13)	*P*-value
BSC	51 (64.6)	41 (82.0)	9 (69.2)	0.102
Treatment	28 (35.4)	9 (18.0)	4 (30.8)	
FOLFOX	12 (14.0)	0	0	
FOLFIRINOX	7 (8.9)	3 (6.0)	0	
S-1	6 (7.6)	0	4 (30.8)	
Gemcitabine + cisplatin	2 (2.5)	2 (4.0)	0	
FOLFIRI	1 (1.3)	0	0	
Clinical trial	1 (1.3)	0	0	
Gemcitabine	0	2 (4.0)	0	
Gemcitabine + nab-paclitaxel	0	1 (1.3)	0	
S-1 + RT	0	1 (1.3)	0	

*NFF* nanoliposomal irinotecan with fluorouracil and folinic acid, *BSC* best supportive care, *RT* radiation therapy.

The majority of patients in the three groups received best supportive care (BSC) alone, including 64.6% of the NFF group, 82.0% of the S-1 group, and 69.2% of the FOLFIRINOX group (P = 0.102). Of those who received subsequent treatment, the most commonly used regimen in theNFF group was FOLFOX (14.0%), followed by FOLFIRINOX (8.9%), S-1 (7.6%), and gemcitabine + cisplatin (2.5%). The most commonly used regimen in the S-1 group, although in a relatively smaller proportion of patients than that in the NFF group, was FOLFIRINOX (6.0%), followed by gemcitabine + cisplatin (4.0%). In the FOLFIRINOX group, 4 patients received subsequent chemotherapy, and all 4 received S-1 monotherapy (30.8%).

## Discussion

In this study, we compared the efficacy of the NFF, S-1, and FOLFIRINOX treatment regimens as second-line treatment for patients with unresectable pancreatic cancer presenting with disease progression after first-line GnP therapy. We demonstrated that the median OS in the NFF group was better than that in the S-1 group, and not significantly different from that in the FOLFIRINOX group; we also identified the serum CRP, serum CA19-9, duration of first-line GnP, and use (yes/no) of S-1 as second-line treatment as variables independently associated with the OS. In addition, the DCR in the NFF group was significantly higher as compared with that in the S-1 group and comparable to that in the FOLFIRINOX group. Furthermore, NFF treatment was not associated with a more favorable OS as compared with S-1 treatment in patient subgroups with serum Alb < 3.5 g/dL and/or serum CRP ≥ 0.3 mg/dL.

We analyzed LAPC and MPC together because in daily clinical practice in Japan, similar regimens are often used for both LAPC and MPC as Clinical Practice Guidelines in Japan stated^15^. We considered that by including LAPC and MPC together, valuable insights into the effectiveness of treatment regimens could be gained across a broader spectrum of patients.

Currently, GnP is widely used as the first-line treatment regimen for patients with unresectable pancreatic cancer, including elderly patients and patients with comorbidities^16,17^. In patients refractory to gemcitabine-based first-line treatment, fluoropyrimidine-based treatments are generally acceptable as second-line treatment. The NAPOLI-1 trial demonstrated the superiority of NFF over 5-FU/LV in patients who were refractory to gemcitabine-based first-line treatment^8^. Based on this, the FDA approved NFF as a subsequent-line treatment option after first-line gemcitabine-based treatment. Besides NFF, sequential treatment with S-1 and FOLFIRINOX are also considered as valid clinical options after failure of first-line gemcitabine-based treatment, however, there is little evidence to suggest the efficacy of these treatments.

Several studies have demonstrated a marginal to favorable efficacy of S-1 in patients with unresectable pancreatic cancer refractory to first-line gemcitabine-based treatment; ORRs ranging from 6 to 27.5%, PFS durations ranging from 1.9 to 4.1 months, and OS durations ranging from 4.5 to 6.9 months have been reported^9,10,18–20^. Based on these results, S-1 is commonly used as a second-line treatment after failure of first-line GnP therapy in patients with unresectable pancreatic cancer in Japan. However, a direct comparison between NFF and S-1 has never been performed. To the best of our knowledge, this study is the first to comparatively evaluate the efficacy of NFF and S-1. In this study, the median OS appears to be better than the median OS of 6.3 months in a randomized phase II study in Japan^21^. Upon comparison, the median CA19-9 level in their study was 1419 U/mL, whereas in our study it was 876 U/mL. This discrepancy suggests a potential presence of more advanced tumors in their study cohort, which may have contributed to the differences in median OS.

In addition, NFF treatment yielded a significantly more favorable DCR, PFS, and OS as compared with S-1. Furthermore, use of S-1 was identified as being independently associated with a poor prognosis, even after adjusting for confounding factors. However, in an exploratory subgroup analysis, among patients with serum Alb < 3.5 g/dL and/or serum CRP ≥ 0.3 mg/dL, NFF treatment was not associated with a more favorable OS as compared with S-1 treatment. This finding underscores the significance of considering the pre-treatment characteristics of the patients, including the aforementioned laboratory data, when making treatment decisions. The rate of treatment discontinuation due to adverse events was 7.1% in the NFF group and 18.2% in the S-1 group among patients with serum Alb < 3.5 g/dL (*P* = 0.20), and 5.0% in the NFF group and 21.1% in the S-1 group among patients with serum CRP ≥ 0.3 mg/dL (*P* = 0.14). One possible explanation for the no significant difference in the rate of treatment discontinuation between the groups was the small number of patients, but these groups with reduced serum albumin and/or elevated serum CRP are considered as being less responsive to chemotherapy. Previous studies have also reported a lower benefit of chemotherapy in patients with low serum Alb and/or high serum CRP levels, and the results of the current study were consistent with these previous reports^22–24^.

The clinical benefit of FOLFIRINOX as second-line treatment after failure of first-line gemcitabine-based treatment has also been investigated. A Korean Cancer Study Group reported similar efficacies between NFF and FOLFIRINOX as second-line treatments in patients with failure of first-line gemcitabine-based treatment, based on the results of a retrospective study^12^. Tezuka et al. also compared these two regimens and reported that while FOLFIRINOX treatment tended to yield a more prolonged survival as compared with NFF treatment (7.4 vs 11.8 months), there was no significant difference in survival between the two treatment groups^25^. While the results of this study should be interpreted with caution due to the small number of patients treated with FOLFIRINOX, our study also found no statistically significant difference between the two treatments. Although a simple comparison cannot be made because of the different studies, we would like to mention that the median OS (4.77 months) in our FOLFIRINOX group was shorter than that reported by Tezuka et al. (11.8 months) and Mie et al. (9.8 months). One possible explanation is the rate of subsequent third-line treatment. In our study, the rate was 30.8%, while Tezuka et al. and Mie et al. had a higher rate of 56.8 and 52.6%, respectively^25,26^. Another possible explanation is that the FOLFIRINOX group in our study had a high proportion of patients with “Duration of first-line GnP < 6 months” (64.3%), which may have included a large number of patients with aggressive tumors. Nevertheless, it is possible that selected patients might benefit from FOLFIRINOX, because a meta-analysis and systematic review have indicated favorable outcomes of platinum-based therapy in patients with homologous recombination deficiency (HRD)^27^. Patients with pancreatic cancer show rapid disease progression, so that it is often difficult to identify HRD prior to the start of the primary treatment and provide treatment accordingly. Therefore, if HRD is identified during or after first-line treatment, therapies such as FOLFIRINOX, which contains platinum agents, could represent a more favorable treatment option.

We showed in this study that apart from the second-line treatment regimen selected, the serum levels of CRP and CA19-9 and duration of first-line GnP therapy are also independent determinants of the OS. Previous studies have revealed elevated serum CRP as a poor prognostic marker in patients with several malignancies^28,29^. Elevated serum CRP has been reported to be associated with various unfavorable prognostic factors, such as a larger tumor size, aggressive histopathological type, and high serum levels of interleukin-6, which plays a crucial role in tumor growth and chemoresistance^30–32^. Serum CA19-9 has also been reported as a prognostic factor in various clinical studies and is considered as a valuable prognostic marker, and its measurement in pancreatic cancer patients is recommended by the NCCN guidelines in a variety of settings, including advanced-stage and perioperative settings^7–34^. Our finding of an association between a shorter duration of first-line GnP treatment and an unfavorable OS is consistent with previous reports^35,11^. Based on the results of this study and other previous studies, closely monitoring patient condition, adjusting chemotherapy dosage as necessary, and implementing comprehensive supportive care might contribute to maximizing the duration of first-line treatment, potentially leading to prolonged OS.

This study had several limitations. Firstly, it was a non-randomized, retrospective integrated analysis of different regimens used in different time interval, which could introduce selection bias and make analysis of the safety profiles difficult. Thus, the patient backgrounds were not well-balanced across the three groups, and we were unable to analyze the safety profiles of the second-line treatments in sufficient number of patients. Further analysis with adjustments for the patient backgrounds, including of the safety profile, is needed. Second, the number of patients treated with FOLFIRINOX included in this analysis was limited. Thus, additional accumulation of patients who have received FOLFIRINOX is necessary to obtain more conclusive findings. Finally, some patients were only clinically diagnosed as having unresectable pancreatic cancer, without histological confirmation. These findings suggest that in real-world settings, some patients may have to undergo systemic chemotherapy without histological evidence for various reasons, including factors related to the patients themselves or the facilities at which they receive treatment.

In conclusion, second-line treatment with NFF was associated with a more favorable OS as compared to that with S-1, although it might be important to consider the patient background characteristics when selecting the treatment for individual patients.

## Methods

### NAPOLEON-1 study

The NAPOLEON-1 study was a multicenter retrospective study conducted with the participation of 14 institutions that compared the efficacy of first-line GnP and FOLFIRINOX in patients with unresectable or recurrent pancreatic cancer in Japan. Between During the period from December 2013 to March 2017, the FOLFIRINOX was initially administered as first-line chemotherapy to 118 patients with unresectable pancreatic cancer, while the GnP regimen was initially given to 200 patients with unresectable pancreatic cancer^13^.

### NAPOLEON-2 study (retrospective part)

The NAPOLEON-2 study (retrospective part) was a multicenter retrospective study conducted with the participation of 25 institutions that evaluated the efficacy of NFF in patients with advanced pancreatic cancer who had a history of systemic therapy in Japan. Between During the period from March 2020 to May 2021, a total of 161 patients were enrolled in this study^11^.

### Patients

This was an integrated analysis of two above-mentioned studies. Data on the following variables were collected from the medical records of the patients: patient demographic characteristics (age and sex), Eastern Cooperative Oncology Group performance status (ECOG PS), tumor histology (adenocarcinoma/non-adenocarcinoma), previous history of tumor resection, sites of metastasis (liver, peritoneum, and/or lung), serum levels of albumin (Alb), C-reactive protein (CRP) and carbohydrate antigen 19-9 (CA19-9), and duration of first-line GnP therapy. Because this study was a retrospective observational study carried out in Japan, informed consent was obtained using the opt-in/opt-out approach according to each participating institution’s policy.

### Treatment

All patients received NFF, S-1, or modified FOLFIRINOX as second-line treatment. Treatment with NFF consisted of intravenous (iv) infusion of Nal-IRI at the dose of 70 mg/m^2^ over 90 min, followed by iv infusion of LV at the dose of 200 mg/m^2^ over 120 min, and then iv infusion of 5-FU at the dose of 2400 mg/m^2^ over 46 h; this treatment was repeated every 2 weeks. If the patients were homozygous for UGT 1A1*6 or *28, or heterozygous for both UGT1A1 *6 and *28 (*UGT1A1*-double variant [DV]), the dose of Nal-IRI was reduced to 50 mg/m^2^^8^. S-1 therapy consisted of oral S-1 administered at the dose of 40 mg/m^2^ twice daily after a meal for 28 consecutive days, followed by a 14 day rest period before repeating the course. The initial dosage was determined based on the patient’s body surface area (BSA): BSA < 1.25 m^2^, 80 mg/day; BSA between 1.25 and 1.50 m^2^, 100 mg/day; and BSA ≥ 1.50 m^2^, 120 mg/day^10^. Treatment with the modified FOLFIRINOX regimen consisted of iv infusion of oxaliplatin at the dose of 85 mg/m^2^ over 2 h, followed by iv infusion of l-leucovorin at the dose of 200 mg/m^2^ over 2 h, followed after 30 min by iv infusion of irinotecan at the dose of 150 mg/m^2^ over 90 min, and finally, continuous iv infusion of 5-fluorouracil at the dose of 2400 mg/m^2^ over a 46 h period; this treatment was repeated every 2 weeks. In patients with *UGT1A1*-DV, the dose of irinotecan was reduced to 120 mg/m^2^ or less^36,37^.

### Assessments

Tumor response was determined according to the Response Evaluation Criteria in Solid Tumors (RECIST) version 1.1^38^. The objective tumor response was assessed by imaging studies, including CT or MRI, in accordance with RECIST version 1.1. An objective response was defined as a complete or partial response, and disease control was defined as a complete response, partial response, or stable disease as the best response. The overall survival (OS) was calculated as the interval from the date of initiation of second-line treatment to the date of death from any cause or date of the last follow-up, or censored at the final follow-up examination. Progression-free survival (PFS) was defined as the time from the date of initiation of second-line treatment to the date of documentation of disease progression or death from any cause, or censored at the final follow-up examination.

### Statistical analysis

Missing data were imputed by using the method of multiple imputation with predictive mean matching^39^. The imputation model included the following variables: serum levels of Alb, CRP, CEA, and CA19-9, and the duration of first-line GnP therapy. However, patients for whom the results of all the blood tests were not available were excluded as cases with insufficient information. The three treatment groups were compared using the Kruskal–Wallis test for continuous data and the chi-square test or Fisher’s exact test for categorical data. We compared the OS and PFS between two groups: (i) the NFF group versus the FOLFIRINOX group, and (ii) the NFF group versus the S-1 group. The p-values were calculated using the unstratified log-rank test, and the hazard ratio (HR) and 95% confidence interval (CI) were determined using unstratified Cox regression. The Cox proportional hazards model was used to identify factors independently associated with the OS. Factors showing differences at *P* < 0.05 were considered as being statistically significant. In subgroup analyses, interaction tests were performed to assess the homogeneity of the effect of treatment on the OS, and the potential for type I error due to multiple comparisons was addressed by adjusting the significance threshold at *P* < 0.05. The statistical analyses were performed using the software program R ver. 4.2.2 (R Foundation for Statistical Computing, Vienna, Austria).

### Ethics approval and consent to participate

This study was conducted in compliance with the ethical guideline outlined in the Declaration of Helsinki and with the central approval of the Institutional review board of Saga Medical Center Koseikan (Study ID 17-09-01-02) and Sasebo Kyosai Hospital (Study ID 2021-08), and also with the approval of the Institutional review boards or ethics committees of the following institutions: National Cancer Center Hospital East, Kagoshima City Hospital, Kagoshima University Graduate School of Medicine and Dental Sciences, Kurume University Hospital, Karatsu Red Cross Hospital, Japanese Red Cross Kumamoto Hospital, University of Miyazaki Hospital, Japanese Red Cross Nagasaki Genbaku Hospital, Japan Community Healthcare Organization Kyushu Hospital, Oita University Faculty of Medicine, Kagoshima Kouseiren Hospital, Saiseikai Kumamoto Hospital, National Hospital Organization Kumamoto Medical Center, Miyazaki Prefectural Miyazaki Hospital, Nagasaki University Hospital, Izumi General Medical Center, Asakura Medical Association Hospital, Saiseikai Sendai Hospital, Hamanomachi Hospital, Imari Arita Kyoritsu Hospital, and Fukuoka Wajiro Hospital. Because this study was a retrospective observational study carried out in Japan, informed consent was obtained using the opt-in/opt-out approach, according to the policies of each participating institution.

### Supplementary Information

Supplementary Table 1.

Supplementary Table 2.

Supplementary Table 3.

## Data Availability

All data generated or analyzed in this article are stored in a secured research database. They are not publicly available, however, they can be made available through the corresponding author upon reasonable request.

## Code availability

We used R ver. 4.2.2 (R Foundation for Statistical Computing, Vienna, Austria) for statistical analyses.
