# Supplementary material for: Nanoliposomal irinotecan with fluorouracil and folinic acid, FOLFIRINOX, and S-1 as second-line treatment for unresectable pancreatic cancer after gemcitabine/nab-paclitaxel
Source: Sci Rep. 2024 Jul 23;14:16906. doi: 10.1038/s41598-024-65689-8 (PMC11266600; doi:10.1038/s41598-024-65689-8)
Supplement: Supplementary file 1 — Supplementary Table 1. [file 41598_2024_65689_MOESM1_ESM.docx]

| Supplementary table 1. Patients characteristics without multiple imputation | | | | |
| --- | --- | --- | --- | --- |
|  | Nal-IRI+5-FU/LV  (n=102) | S-1  (n=57) | FOLFIRINOX  (n=14) | *P*-value |
| Age (years), *n* (%) |  |  |  |  |
| <70 | 52 (51.0) | 32 (56.1) | 10 (71.4) | 0.335 |
| ≥70 | 50 (49.0) | 25 (43.9) | 4 (28.6) |  |
| Sex (M/F), *n* (%) |  |  |  |  |
| Male | 61 (59.8) | 34 (59.6) | 9 (64.3) | 0.946 |
| Female | 41 (40.2) | 23 (40.4) | 5 (35.7) |  |
| ECOG PS, *n* (%) |  |  |  |  |
| 0 | 49 (48.0) | 16 (28.1) | 7 (50.0) | 0.040 |
| 1 or more | 53 (52.0) | 41 (71.9) | 7 (50.0) |  |
| Histology, *n* (%) |  |  |  |  |
| Adenocarcinoma | 93 (91.2) | 50 (87.7) | 13 (92.9) | 0.513 |
| Others^a^ | 5 (4.9) | 0 | 0 |  |
| Unknown | 4 (3.9) | 7 (12.3) | 1 (7.1) |  |
| Prior pancreatectomy, *n* (%) | 14 (13.7) | 5 (8.8) | 3 (12.4) | 0.397 |
| Disease extension, *n* (%) |  |  |  |  |
| Locally advanced | 12 (11.8) | 14 (24.6) | 2 (14.3) | 0.108 |
| Metastatic | 90 (88.2) | 43 (75.4) | 12 (85.7) |  |
| Metastatic site, *n* (%) |  |  |  |  |
| Liver | 65 (63.7) | 22 (38.6) | 6 (42.9) | 0.007 |
| Lung | 16 (15.7) | 10 (17.5) | 2 (14.3) | 0.935 |
| Peritoneal | 28 (27.5) | 22 (38.6) | 5 (35.7) | 0.332 |
| Ascites, *n* (%) | 18 (17.6) | 10 (17.5) | 2 (14.3) | 0.951 |
| Albumin (g/dL), *n* (%) |  |  |  |  |
| ≥3.5 | 58 (56.8) | 30 (52.6) | 7 (50.0) | 0.973 |
| <3.5 | 42 (41.2) | 20 (35.1) | 5 (35.7) |  |
| Missing | 2 (2.0) | 7 (12.3) | 2 (14.3) |  |
| CRP (mg/dL), *n* (%) |  |  |  |  |
| <0.3 | 42 (41.2) | 18 (17.5) | 5 (35.7) | 0.603 |
| ≥0.3 | 59 (57.8) | 36 (63.2) | 8 (57.1) |  |
| Missing | 1 (1.0) | 3 (5.3) | 1 (7.1) |  |
| CA19-9 (U/mL), *n* (%) |  |  |  |  |
| <1000 | 48 (47.1) | 29 (50.9) | 10 (71.4) | 0.085 |
| ≥1000 | 54 (52.9) | 21 (36.8) | 3 (21.4) |  |
| Missing | 0 (0) | 7 (12.3) | 1 (7.1) |  |
| Duration of first-line GnP, n (%) |  |  |  |  |
| ≥6 | 60 (58.8) | 39 (68.4) | 5 (35.7) | 0.077 |
| <6 | 36 (35.3) | 18 (31.6) | 9 (64.3) |  |
| Missing | 6 (5.9) | 0 (0) | 0 (0) |  |
| UGT1A1 status |  |  |  |  |
| -/- | 55 | 0 | 4 |  |
| -/*6 | 22 | 1 | 2 |  |
| -/*28 | 16 | 1 | 3 |  |
| *6/*6 | 3 | 0 | 1 |  |
| *6/*28 | 2 | 3 | 1 |  |
| Unknown | 4 | 52 | 3 |  |
| Treatment cycles, median (range) | 5 (1 - 38) | - | - |  |
| Treatment duration (days), median (range) | 83 (2 - 568) | - | - |  |
| \| *Abbreviations:* *Nal-IRI+5-FU/LV*, nanoliposomal irinotecan with fluorouracil and folinic acid; *ECOG PS,* Eastern Cooperative Oncology Group performance status; serum *CRP,* C-reactive protein; serum *CA19-9,* carbohydrate antigen 19-9; *GnP*, Gemcitabine plus nab-paclitaxel  ^a^ acinar cell carcinoma, adenosquamous carcinoma and intrapapillary mucinous carcinoma \| \| --- \| | | | | |
