# Supplementary material for: Nanoliposomal irinotecan with fluorouracil and folinic acid, FOLFIRINOX, and S-1 as second-line treatment for unresectable pancreatic cancer after gemcitabine/nab-paclitaxel
Source: Sci Rep. 2024 Jul 23;14:16906. doi: 10.1038/s41598-024-65689-8 (PMC11266600; doi:10.1038/s41598-024-65689-8)
Supplement: Supplementary file 2 — Supplementary Table 2. [file 41598_2024_65689_MOESM2_ESM.docx]

| **Supplementary table 2. Univariate and multivariate analyses conducted using Cox proportional hazards models to predict survival in patients with unresectable pancreatic cancer.** | | | | | | | | | |
| --- | --- | --- | --- | --- | --- | --- | --- | --- | --- |
| Variables | Univariate analysis | | |  | Multivariate analysis | | | | |
|  | HR | 95% CI | *P*-value |  | HR | 95% CI | | | *P*-value |
| Age |  |  |  |  |  | |  |  | |
| <70 years | reference |  |  |  | reference | |  |  | |
| ≥70 years | 0.937 | 0.628 – 1.399 | 0.751 |  | 1.113 | | 0.675 – 1.834 | 0.674 | |
| Sex |  |  |  |  |  | |  |  | |
| Female | reference |  |  |  | reference | |  |  | |
| Male | 0.967 | 0.645 – 1.448 | 0.871 |  | 0.721 | | 0.447 – 1.162 | 0.179 | |
| ECOG PS |  |  |  |  |  | |  |  | |
| 0 | reference |  |  |  | reference | |  |  | |
| 1 or more | 1.876 | 1.225 – 2.874 | 0.004 |  | 1.624 | | 0.976 – 2.702 | 0.062 | |
| Prior pancreatectomy: Yes | 0.819 | 0.425 – 1.579 | 0.552 |  | 0.596 | | 0.271 – 1.308 | 0.197 | |
| Metastases: Yes | 0.934 | 0.552 – 1.581 | 0.800 |  | 1.398 | | 0.757 – 2.581 | 0.285 | |
| Albumin |  |  |  |  |  | |  |  | |
| ≥3.5 g/dL | reference |  |  |  | reference | |  |  | |
| <3.5 g/dL | 1.920 | 1.251 – 2.943 | 0.003 |  | 1.329 | | 0.827 – 2.134 | 0.240 | |
| CRP |  |  |  |  |  | |  |  | |
| <0.3 mg/dL | reference |  |  |  | reference | |  |  | |
| ≥0.3 mg/dL | 3.360 | 2.050 – 5.500 | <0.001 |  | 3.319 | | 1.837 – 5.996 | <0.001 | |
| CA19-9 |  |  |  |  |  | |  |  | |
| <1000 U/mL | reference |  |  |  | reference | |  |  | |
| ≥1000 U/mL | 1.770 | 1.160 – 2.691 | 0.008 |  | 1.920 | | 1.168 – 3.157 | 0.010 | |
| Duration of first-line GnP |  |  |  |  |  | |  |  | |
| ≥6 months | reference |  |  |  | reference | |  |  | |
| <6 months | 1.960 | 1.300 – 2.940 | 0.001 |  | 2.794 | | 1.714 – 4.554 | <0.001 | |
| Second-line treatment |  |  |  |  |  | |  |  | |
| Nal-IRI+5-FU/LV | reference |  |  |  | reference | |  |  | |
| S-1 | 1.966 | 1.283 – 3.011 | 0.002 |  | 3.065 | | 1.794 – 5.239 | <0.001 | |
| FOLFIRINOX | 1.271 | 0.650 – 2.484 | 0.484 |  | 1.335 | | 0.578 – 3.083 | 0.498 | |
| *Abbreviations: HR*, hazard ratio; *CI*, confidence interval; *ECOG PS,* Eastern Cooperative Oncology Group performance status; *LDH,* lactate dehydrogenase; *CRP,* serum C–reactive protein, serum *CA19-9* carbohydrate antigen 19-9; *GnP*, Gemcitabine plus nab-paclitaxel; *Nal-IRI+5-FU/LV*, nanoliposomal irinotecan with fluorouracil and folinic acid | | | | | | | | | |
