# Supplementary material for: Nanoliposomal irinotecan with fluorouracil and folinic acid, FOLFIRINOX, and S-1 as second-line treatment for unresectable pancreatic cancer after gemcitabine/nab-paclitaxel
Source: Sci Rep. 2024 Jul 23;14:16906. doi: 10.1038/s41598-024-65689-8 (PMC11266600; doi:10.1038/s41598-024-65689-8)
Supplement: Supplementary file 3 — Supplementary Table 3. [file 41598_2024_65689_MOESM3_ESM.docx]

|  | Supplementary table 3. Grade 3 or higher adverse events of second-line nanoliposomal irinotecan with fluorouracil and folinic acid | | | | | |
| --- | --- | --- | --- | --- | --- | --- |
| Event | | | All  (n=98) | Wild type  (n=55) | UGT1A1 single hetero  (n=38) | UGT1A1 double hetero/homo  (n=5) |
| Hematological, n (%) | | **Leukopenia** | 11 (11.2) | 2 (3.6) | 8 (21.1) | 1 (20.0) |
|  | | **Neutropenia** | 22 (22.4) | 8 (14.5) | 13 (34.2) | 1 (20.0) |
|  | | **Anemia** | 9 (9.2) | 6 (10.9) | 2 (5.3) | 1 (20.0) |
|  | | **Thrombocytopenia** | 1 (1.0) | 0 (0) | 1 (2.6) | 0 (0) |
| Non-hematological, n (%) | | **Febrile neutropenia** | 1 (1.0) | 0 (0) | 1 (2.6) | 0 (0) |
|  | | **Anorexia** | 10 (10.2) | 5 (9.1) | 5 (13.2) | 0 (0) |
|  | | **Diarrhea** | 4 (4.1) | 2 (3.6) | 2 (5.3) | 0 (0) |
|  | | **Nausea** | 3 (3.1) | 0 (0) | 0 (0) | 0 (0) |
|  | | **Vomiting** | 1 (1.0) | 0 (0) | 1 (2.6) | 0 (0) |
|  | | **Sensory neuropathy** | 1 (1.0) | 0 (0) | 0 (0) | 0 (0) |
|  | | **AST/ALT increased** | 1 (1.0) | 0 (0) | 1 (2.6) | 0 (0) |
|  | | **Biliary tract infection** | 3 (3.1) | 1 (1.8) | 2 (5.3) | 0 (0) |
|  | | **Fatigue** | 2 (2.0) | 0 (0) | 1 (2.6) | 0 (0) |
|  | | **Rash** | 1 (1.0) | 0 (0) | 1 (2.6) | 0 (0) |
|  | | **thromboembolism** | 1 (1.0) | 0 (0) | 1 (2.6) | 0 (0) |
|  | | **Abdominal pain** | 1 (1.0) | 1 (1.8) | 0 (0) | 0 (0) |
